# Editorial: Integrating Timescales From Molecules Up

**DOI:** 10.3389/fchem.2021.680533

**Published:** 2021-04-13

**Authors:** Rene A. Nome, Pilar Cossio, Y Z

**Affiliations:** ^1^Department of Physical Chemistry, Institute of Chemistry, State University of Campinas - Unicamp, Campinas, Brazil; ^2^Biophysics of Tropical Diseases, Max Planck Tandem Group, University of Antioquia UdeA, Medellín, Colombia; ^3^Department of Theoretical Biophysics, Max Planck Institute of Biophysics, Frankfurt am Main, Germany; ^4^Department of Nuclear, Plasma, and Radiological Engineering, Department of Electrical and Computer Engineering, Beckman Institute for Advanced Science and Technology, University of Illinois at Urbana-Champaign, Urbana, IL, United States

**Keywords:** rare events, long timescale processes, ultrafast, barrier crossing, single molecule

Barrier crossing in thermally activated processes is a fundamental and ubiquitous concept in Chemistry, Physics, and Biology. For example, in the study of reaction mechanisms, chemical kinetics investigations enable the determination of rate laws and the elucidation of elementary steps, while femtochemistry has sufficient time resolution to characterize the transition state and reaction intermediates. Despite the large timescale difference between typical reaction rate-constants and transition path lifetimes, both are crucial for understanding reaction mechanisms. Therefore, ideally one would like to integrate, conceptually and experimentally, the methods used at different timescales in order to obtain a more complete picture of how and why reactions occur. Examples where this general strategy can be applied abound, including biomolecular folding, catalysis, glass transition, nucleation and crystal growth, molecular and colloidal self-assembly, and among others.

Although timescale integration is a simple and well-known idea, and despite current efforts, it is still challenging to provide systematic structure-reactivity trends for general chemical and biological systems from this approach. This is not entirely surprising since usually the tools employed to study fast and slow thermally-activated processes are fundamentally different: stroboscopic and asynchronous methods, respectively.

This Frontiers in Chemistry, Frontiers in Physics, and Frontiers in Molecular Biosciences focus topic “*Integrating Timescales from Molecules Up*” tackles the challenges of studying barrier-crossing events from a wide time-scale perspective. We highlight contributions from multiple fields of research employing a growing class of interdisciplinary experimental, computational, and theoretical tools.

Drawing insights from scattering experiments and molecular dynamics simulations of metallic liquids, Egami and Ryu suggest that the noted over 15 orders of magnitude change in viscosity when liquids freeze into glasses can be caused by relatively small changes in the structural coherence in the medium range. Hu et al. use persistent homology to study quantum effects—delocalization, zero-point energy, and tunneling—in glassy dynamics and proton transfer reactions from a computational-topology perspective. The contribution from Sadighian et al. exemplifies how to integrate single-shot femtosecond transient absorption spectroscopy with *in situ* and real-time studies of perovskite nanocrystal nucleation and growth, which occurred on the time-scale from a few minutes to a couple hours. Kim et al. study push-pull chromophore polymers and show how the nature of the linker covalent bond (sigma vs. pi) influences photoinduced energy transfer dynamics on the nanosecond to microsecond timescales. Datta et al. study open system dynamics during the laser-induced creation of quasi-stationary states in mixtures of ionic liquids with liquid crystals at the nematic-isotropic phase transition.

On the biological side, Mehlich et al. use single-molecule force-spectroscopy experiments to characterize the slow transition-path time, on the order of milliseconds, of a *de-novo* designed protein. Reaching similar time scales, but from a computational perspective, Burke et al. use discrete path sampling to characterize the metastable and transition states in the membrane fusion pathway of Influenza A Hemagglutinin. Scull et al. highlight the functional importance of timescale integration from microseconds to minutes in riboswitches, encompassing ligand binding, RNA folding and transcription, and bacterial gene expression control.

Overall, this Research Topic highlights the utility of learning ideas from different fields, and suitably adapting them to the study of a system's dynamics. As stated above, recent advances have come from the integration of experimental tools such as ultrafast time-resolved non-linear laser spectroscopy, optical microscopy, single-molecule spectroscopy, neutron, and X-ray scattering, as well as theoretical and computational methods such as non-equilibrium statistical mechanics, stochastic dynamics, and rare event sampling. These advances may impact the study of biophysics, disordered materials, nanomaterials, catalysis, colloid science, and among others.

## Author Contributions

All authors contributed to manuscript writing and revision, read, and approved the submitted version.

## Conflict of Interest

The authors declare that the research was conducted in the absence of any commercial or financial relationships that could be construed as a potential conflict of interest.

